# Supplementary material for: Unraveling the genetic architecture of congenital vertebral malformation with reference to the developing spine
Source: Nat Commun. 2024 Feb 6;15:1125. doi: 10.1038/s41467-024-45442-5 (PMC10847475; doi:10.1038/s41467-024-45442-5)
Supplement: Supplementary file 1 — Supplementary Information [file 41467_2024_45442_MOESM1_ESM.pdf]

Unraveling the genetic architecture of congenital vertebral malformation with reference to the developing spine

Sen Zhao[1,2,3], Hengqiang Zhao[1,4,5], Lina Zhao[1,4,6], Xi Cheng[1], Zhifa Zheng[1,4,5], Mengfan Wu[7], Wen Wen[1], Shengru Wang[1], Zixiang Zhou[1], Haibo Xie[7], Dengfeng Ruan[8], Qing Li[1,4,5], Xinquan Liu[1], Chengzhu Ou[1], Guozhuang Li[1,4,5], Zhengye Zhao[1,4,5], Guilin Chen[1,4,5], Yuchen Niu[2,4,5,6], Xiangjie Yin[1,4,5], Yuhong Hu[1], Xiaochen Zhang[1], Deciphering disorders Involving Scoliosis and COmorbidities study[#], Pengfei Liu[4,9], Guixing Qiu[1,2,4,5], Wanlu Liu[8], Chengtian Zhao[7], Zhihong Wu[2,4,5,6]*, Jianguo Zhang[1,2,4,5]*, and Nan Wu[1,2,4,5]*

Contents

Supplementary Methods
Supplementary Tables 1-5
Supplementary Figures 1-9
Supplementary References

Supplementary Methods

Next generation sequencing

Exome sequencing (ES)

Illumina paired-end libraries were prepared from peripheral blood DNA samples. Five different capture kits were used: xGen Exome Research Panel v1/v2 (IDT, Coralville, IA USA), SeqCap EZ HGSC VCRome (Roche, Pleasanton, CA, USA), NimbleGen SeqCap EZ Exome (Roche, Pleasanton, CA, USA), KAPA HyperExome (Roche, Pleasanton, CA, USA), and Agilent SureSelect Human All Exon V5/V6/V6+UTR/V7 (Agilent, Santa Clara, CA, USA) (**Supplementary Table 2**). The sequencing run was performed in paired-end mode using the Illumina (Illumina, San Diego, CA, USA) HiSeq 2000 platform.

Genome sequencing (GS)

For GS, sequencing libraries were prepared using the KAPA Hyper Prep kit (KAPA Biosystems, Kusatsu, Japan) according to the optimized manufacturer's protocol. Multiplex sequencing was performed using an Illumina (Illumina, San Diego, CA, USA) HiSeq X-Ten or NovaSeq platform.

Sequence data processing and quality control

Raw sequencing reads were mapped to hg19 human reference genome using Picard (broadinstitute.github.io/picard/) and BWA[1]. Single-nucleotide variants (SNVs) and small insertions/deletions (indels) were called using the DNAseq module of the Sentieon software[2]. SNVs and indels underwent multiple layers of filtration:

Hard filter: We filtered out variants that met any of the following criteria:

- genotype quality (GQ) < 20
- depth (DP) < 10
- quality by depth (QD) < 2
- strand odds ratio > 9
- Variant allele balance < 25%

Population-based filter: We filtered out variants that deviated from the Hardy-Weinberg equilibrium ($p < 10^{-6}$) and variants with a missing rate > 10% in the case-control population.

Variant Quality Score Recalibration (VQSR) was performed using the standard GATK protocol with a sensitivity of 99%.

Small insertions/deletions (indels) located in low-complexity repeat regions (RepeatMasker.bed retrieved from the UCSC genome browser) were filtered out.

After variant-level QC, we performed ancestry estimation by principal component analysis (PCA) using plink (version 1.90) based on the combined genotypes of the in-house subjects and the 1000 Genomes Phase III population (**Supplementary Fig. 9**). Samples that met any of the following criteria were excluded:

- PCA outliers (outside ±3 standard deviations)
- Overall call rate < 0.8
- Average depth < 20X
- Heterozygosity (< 3 standard deviations)

In addition, the relatedness among individuals was calculated using the identity-by-descent (IBD) analysis. For each pair of individuals with IBD > 0.8, we excluded the one with a lower call rate. The numbers of samples filtered out due to each criterion were shown in **Supplementary Table 4.**

Variant annotation

The effects of variants on the transcript and the protein sequence were annotated with the ENSEMBL variant effect predictor (VEP, version 104)[3]. The LOFTEE (https://github.com/konradjk/loftee) and dbNSFP [4] (v4.3a) plugins were used to generate bioinformatic predictions. The Genome Aggregation Database (gnomAD, https://gnomad.broadinstitute.org/, v2.1.1, accession date: 2022/05/25) was used to annotate population frequencies for the variants.

For individuals with both parents sequenced (i.e., trio families), *de novo*, compound heterozygous, and recessively inherited variants were annotated using slivar (https://github.com/brentp/slivar).

Variant interpretation

We first filtered out common variants (gnomAD MAF > 5%) which are likely benign. The pathogenicity of retained variants was then evaluated according to the American College of Medical Genetics and Genomics/the Association for Molecular Pathology (ACMG/AMP) guidelines [5]. In brief, the variant type, population frequency, functional results, inheritance origin, and reported cases were considered as evidence of various supporting levels. Collected evidence was evaluated in aggregation to come up with a pathogenicity classification, including 'pathogenic', 'likely pathogenic', 'variant of uncertain significance', 'likely benign', and 'benign' [5].

The anticipated mode of inheritance associated with the identified genes was then considered. For genes whose variants showed an autosomal dominant or X-linked inheritance pattern, a heterozygous variant was sufficient to be potentially disease-causing. For genes whose variants showed an autosomal recessive inheritance mode, biallelic variants revealed through trio exome sequencing were required to suspect a gene to be disease-causing.

If the observed variant(s) was/were pathogenic and consistent with the expected mode of inheritance, gene-related phenotypes were compared to the patient phenotype. A molecular diagnosis was made if the phenotypic spectrum of the gene could explain the whole clinical spectrum of the patient.

Calling copy number variants (CNVs) from ES and GS data

Copy number variants (CNVs) were called using XHMM[6] (for ES data) and CNVnator[7] (for GS data), both of which are read depth-based methods. For XHMM, exome data were divided into batches depending on the capture kit used. Exome coverage data from each batch were jointly analyzed. Raw XHMM calls were filtered using the standard protocol[8]. For CNVnator, each genome data was analyzed independently. Raw CNV calls were merged and filtered according to an established protocol[9]. All retained CNVs were annotated using the annotSV tool and ready for clinical interpretation[10,11].

Interpretation of CNVs

A CNV was firstly compared with the recurrent CNV region reported in the Database of Chromosomal Imbalance and Phenotype in Humans using Ensembl

Resources [12] (DECIPHER community, https://decipher.sanger.ac.uk/, accession date 2022-02-14). We defined a CNV as a recurrent pathogenic CNV by using the threshold for reciprocal overlap >80% with the reported CNVs. The remaining CNVs (<80% overlap with reported CNVs) were checked in the Database of Genomic Variants (DGV, http://dgv.tcag.ca/dgv/app/home) and the segmental duplication regions. We defined a CNV as benign if it is recorded with a frequency >1% in the DGV database and/or with an overlap >80% in the segmental duplication regions.

Construction of *alpk3a* and *alpk3b* double knock-out (DKO) zebrafish model

We used the CRISPR-Cas9 system to generate *alpk3a* and *alpk3b* double knock-out (DKO) zebrafish. gRNA sequences used are as follows:

Gene (target)	gRNA
alpk3a (exon2)	AGAGCGGGTCATGGGCCTCC
alpk3b (exon3)	AAATGGAAGAAGGGAAGAGA;<br>AAGAGATGGTGATTGAGAGT

Co-injection of two gRNA targeting alpk3b were performed for better efficiency. gRNA sequences were compared against zebrafish genome and transcriptome using ensemble BLAST (ensembl.org) to control for off-target effects.

Genotyping of *alpk3a* and *alpk3b* frameshift variants were performed using the following primers:

Gene (variant)	Primers
alpk3a (-6+20bp at exon2)	F:GGACTGTTGAAGTTGTTTTGT<br>R:ATCCTCCCCATTTTGGTTTC
alpk3b (-20bp at exon3)	F:AAACAATGCCACAAGTGAAC<br>R:CAGTCTCTGAAACCATCTGT

Construction of *Alpk3*$^{-/-}$ mouse model

Mice used in the study were housed under controlled environmental conditions. The animal facility maintained a consistent 12-hour light/12-hour dark cycle, with lights on at 07:00 AM and off at 07:00 PM. The ambient temperature in the housing

area was consistently maintained at 22 ± 2°C. Relative humidity was maintained at 50 ± 10% throughout the study period.

We used the CRISPR-Cas9 system to delete exon3 of *Alpk3* in C57BL/6 background. gRNA sequences used are as follows:

Gene (target)	gRNA
Alpk3 (5’ side of exon3)	CCATGTCTGTCAGTCACCTG
Alpk3 (3’ side of exon3)	TGTTGACATGGTCTCTATCA

gRNA sequences were compared against zebrafish genome and transcriptome using ensemble BLAST (ensembl.org) to control for off-target effects.

Genotyping of exon3 deletion was performed using the following primer:

Gene (variant)	Primer
Alpk3 (exon3 deletion)	F:CAGCTCCCCACCATCACGCAGTAA<br>R:TGGGGCTTCATTGTCTTTGTTTTC

Demineralized murine skeletal histology

For H&E staining, demineralized sections underwent deparaffinization in xylene and rehydration through a descending ethanol series to water. Hematoxylin staining was applied for a specific duration, followed by washing and counterstaining with eosin. After a final wash, sections were dehydrated in an ascending ethanol series, cleared in xylene, and mounted with coverslips using a mounting medium. Stained sections were examined under a light microscope. Hematoxylin provided blue-purple nuclear staining, while eosin stained the cytoplasm and extracellular matrix in varying shades of pink. Staining times and conditions were adjusted based on tissue size and density, with regular microscopic checks to ensure staining quality.

For Toluidine blue staining, demineralized sections were fixed in paraformaldehyde and then embedded in paraffin following standard dehydration and clearing procedures. Sections of 4-6 μm thickness were cut using a microtome and mounted on glass slides. Prior to staining, sections were deparaffinized in xylene and rehydrated through a graded series of ethanol to water. The slides were then stained with 0.1% Toluidine Blue solution for two minutes. The stain was prepared in a mildly acidic solution (pH 2.3-4.5) to enhance the contrast. After staining, the

slides were briefly rinsed in distilled water to remove excess stain, and then dehydrated quickly through an ascending series of ethanol. Following dehydration, sections were cleared in xylene and mounted with a coverslip using a mounting medium.

Single-nucleus RNA sequencing (snRNA-seq) of the human embryonic spine

Human spine tissues were subjected to a rigorous washing process with cold and sterilized PBS twice, and then transported to the laboratory in MACS® Tissue Storage Solution (Miltenyi Biotec). Upon arrival at the laboratory, the spine tissues were isolated and rapidly frozen in liquid nitrogen for preservation.

The frozen tissues were then mixed with 700-800 μL of lysis buffer (LB), consisting of 10 mM Tris-HCl, 10 mM NaCl, 3 mM $MgCl_2$, 0.1% Tween-20, 0.1% Nonidet P40, 0.01% Digitonin, 1 mM DTT, 1 U/μL RNase inhibitor, and 1% BSA, and placed on ice for 2-5 minutes for lysis. After completion of lysis, an equal volume of wash buffer (10 mM Tris-HCl, 10 mM NaCl, 3 mM $MgCl_2$, 0.1% Tween-20, 1 mM DTT, 1 U/μL RNase inhibitor, and 1% BSA) was added to terminate the lysis. The lysate was then purified using a 40 μm cell strainer (Becton, Dickinson and Company), transferred to a new 5mL tube, and centrifuged for 5 minutes at 4 °C and 500 g. The sediment was retained and mixed with 300 μL LB solution, resulting in single nucleus suspensions. Single nucleus suspensions with a concentration of 10000-20000 cells/mL in PBS were then processed for library preparation following the 10X Genomics standard protocol (CG000338). The snRNA libraries were subsequently sequenced on the Illumina HiSeq X platform using 150 bp paired-end reads.

snRNA-seq data analyses

The Cell Ranger pipeline version 7.0 was used for the alignment of raw sequences to the human reference genome hg38. GENCODE v32 was used as a reference to generate a single cell-gene expression matrix for each sample. Quality control of cells was performed based on their expression signatures. We also performed doublet prediction using Doublet Finder v2 (PCs = 1:30; pN = 0.25; pK = 0.01) and Scrublet v 0.1 (expected_doublet_rate = 0.06; min_counts = 2;

min_cells = 3; min_gene_variability_pctl = 85, n_prin_comps = 30). We filtered out cells that reached any of the following criteria:

- Mitochondrial reads > 5%
- Number of unique genes detected < 1000
- Involved in cell doublets or multiplets

The expression matrix from each sequenced sample underwent normalization, scalation, and selection of variable features through Seurat (https://github.com/satijalab/seurat, v4.2) [13]. Data from five different samples were then merged using the Seurat integration function. After data integration, we performed principal component analysis (PCA) linear dimensionality reduction on the scaled data and clustered the cells with a graph-based clustering approach (RunPCA). Thirty principal components were retained and used to construct a k-nearest neighbors graph based on the Euclidean distance in PCA space. We then refined the edge weights between any two cells based on the shared overlap in their local neighbors (FindNeighbors, dims = 1:30). We then clustered the cells using the Louvain algorithm (in Seurat) to iteratively group cells together while optimizing the standard modularity function (FindClusters, algorithm = 1, method = "matrix").

To annotate clusters, we determined differentially expressed genes using FindAllMarkers from Seurat (Wilcoxon Rank Sum test with Bonferroni correction for multiple testing; adjusted $P < 0.05$). By reviewing the differentially expressed genes as well as the expression of canonical marker genes involved in the developing spine, we assigned a cell-type identity to each cell cluster.

Supplementary Tables

Supplementary Table 1. Intraspinal malformations and sex differences

**Malformation**[a]	**N male (%)**	**N female (%)**	**P value**[b]
Intraspinal malformation	63 (14.8)	123 (27.5)	7.84E-04
Diastomyelia	40 (9.4)	95 (21.2)	9.97E-05
Syringomyelia	30 (7.1)	51 (11.4)	6.43E-02
Tethered spinal cord	15 (3.5)	32 (7.1)	3.81E-02

[a] the numbers may not add up to the sum because there are patients with multiple intraspinal malformation.

[b] P-values were calculated using the Chi-square test.

Supplementary Table 2. Exome sequencing (ES) and genome sequencing (GS) on families with congenital vertebral malformation (CVM) and control samples

Sequencing method and capture	CVM cohort (probands)	CVM cohort (familial members)	Control cohort
Exome sequencing	262	293	2890
Genome sequencing	611	212	904
Sum	873	505	3794

Supplementary Table 3. Occurrence of vertebral fusion in *Alpk3*$^{-/-}$ or wt mice at different ages

	WT			*Alpk3*$^{-/-}$			p-value[a]
	**N**	**Vertebral fusion**	**Prevalence**	**N**	**Vertebral fusion**	**Prevalence**	
P0	8	0	0.00	14	0	0.00	-
3W	18	0	0.00	12	2	0.17	0.2139
5W	16	0	0.00	16	7	0.44	0.0034
8W	20	2	0.10	12	8	0.67	0.00153
10W+	15	1	0.07	29	19	0.66	0.00018

[a] Occurrence of vertebral malformation between KO and wt groups were tested using Fisher's Exact test.

Abbreviation: WT, wide-type.

Supplementary Table 4. Sample-level quality control

Criteria	N case samples removed	N control samples removed
Low overall depth	3	13
High missing rate	24	39
Low heterozygosity	1	4
Hight relatedness	2	14
PCA outlier	2	0
**Sum[a]**	**29**	**54**

[a] the numbers may not add up to the sum because there are samples that failed multiple quality control (QC) criteria.

Supplementary Table 5. Variant weighting criteria in the gene-based burden analysis

**Weight [a]**	**Loss-of-function variants[b]**	**In-frame indels**	**Missense variants**
1	Labeled as 'high confidence' by LOFTee	-	-
0.8	Labeled as 'low confidence' or unlabeled by LOFTee	-	REVEL > 0.8
0.5	SpliceAI > 0.5	CADD score > 20	REVEL > 0.6
0.3	-	CADD score > 10	REVEL > 0.4
0.1	-	Other	REVEL > 0.2
0	-	-	Other

[a] For a variant that fulfilled the criteria of more than one weight score level, the maximum weight score was used.

b Loss-of-function variants include nonsense variants, frameshift variants, canonical splicing variants, and start-loss variants.

Abbreviations: CADD, Combined Annotation Dependent Depletion(a tool for scoring the deleteriousness of single nucleotide variants as well as insertion/deletions variants in the human genome); REVEL, Rare Exome Variant Ensemble Learner (an ensemble method for predicting the pathogenicity of missense variants); indel, insertion/deletion.

Supplementary figures and legends

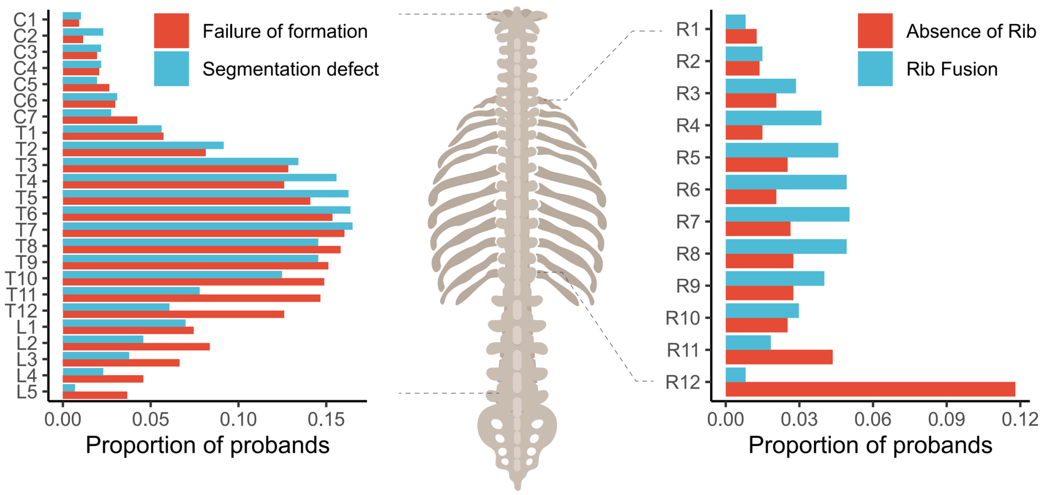

Supplementary Fig. 1 Characteristic of vertebral malformation and rib malformation in the cohort

The proportion of two types of vertebral malformation (failure of formation and segmentation defect) of cervical/thoracic/lumbar vertebrae and the proportion of two different types of rib malformation (absence of rib and rib fusion) of twelve ribs in all probands are shown (n=873). C, cervical vertebrae; T, thoracic vertebrae; L, lumbar vertebrae; R, rib.

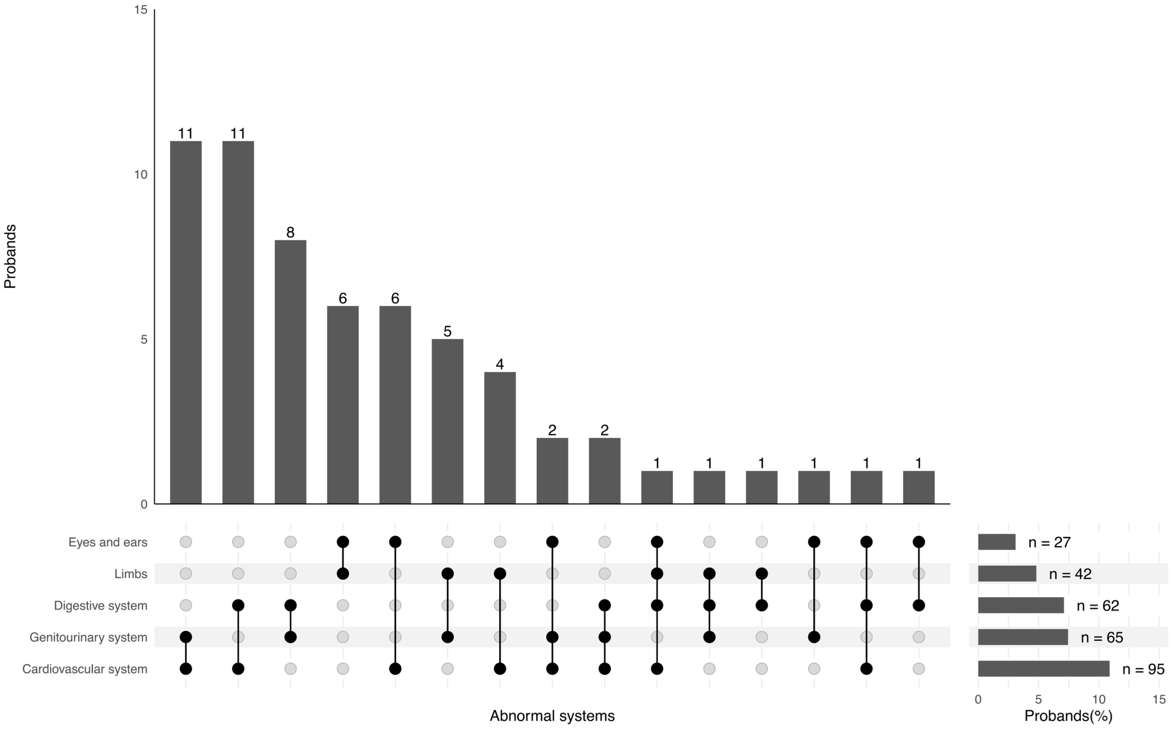

Supplementary Fig. 2 Characteristics of systemic malformations and comorbidities

Distribution of probands with extra-spinal defects in eyes and ears, limbs, digestive system, genitourinary system, and cardiovascular system, and co-occurrence of these anomalies.

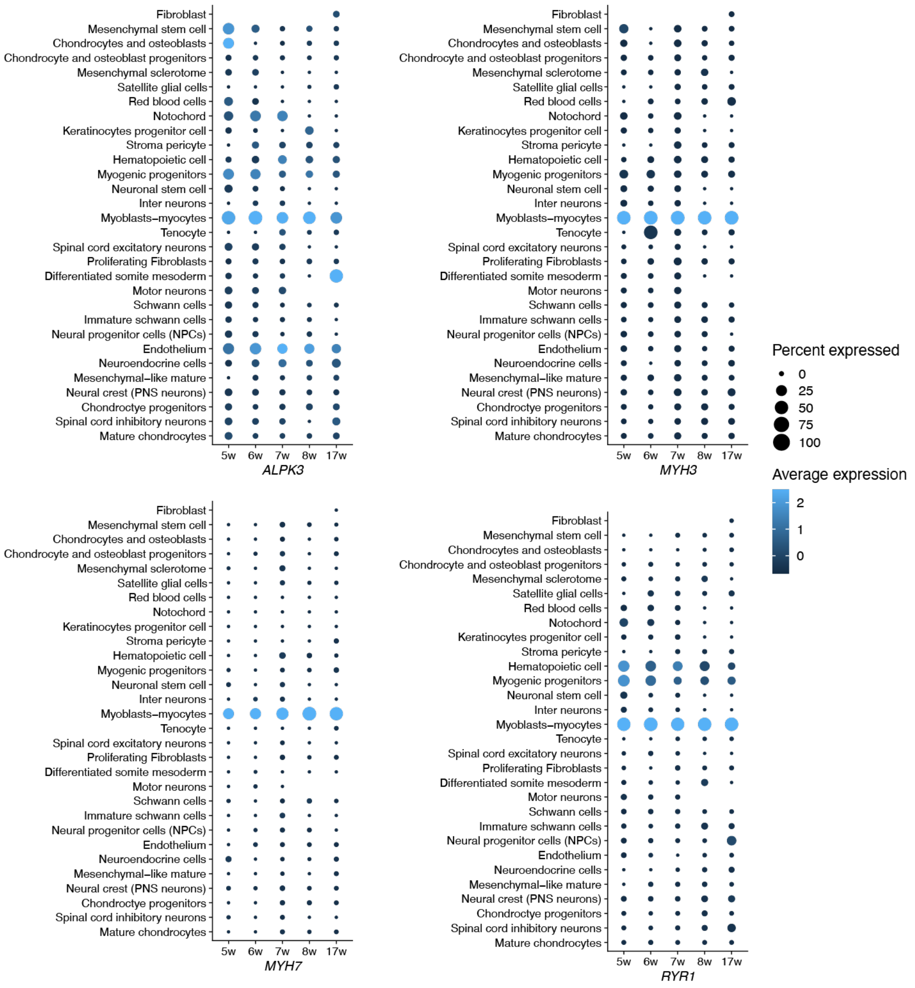

Supplementary Fig. 3 Expression of four muscle-related genes in the developing spine

Expression of four muscle-related genes (*ALPK3*, *MYH3*, *MYH7*, *RYR1*) in different cell types in human embryonic spine along gestation stages. Percent expressed indicates the percent of cells expressing the gene in each cell type. The average expression indicates the mean normalized gene expression in each cell type.

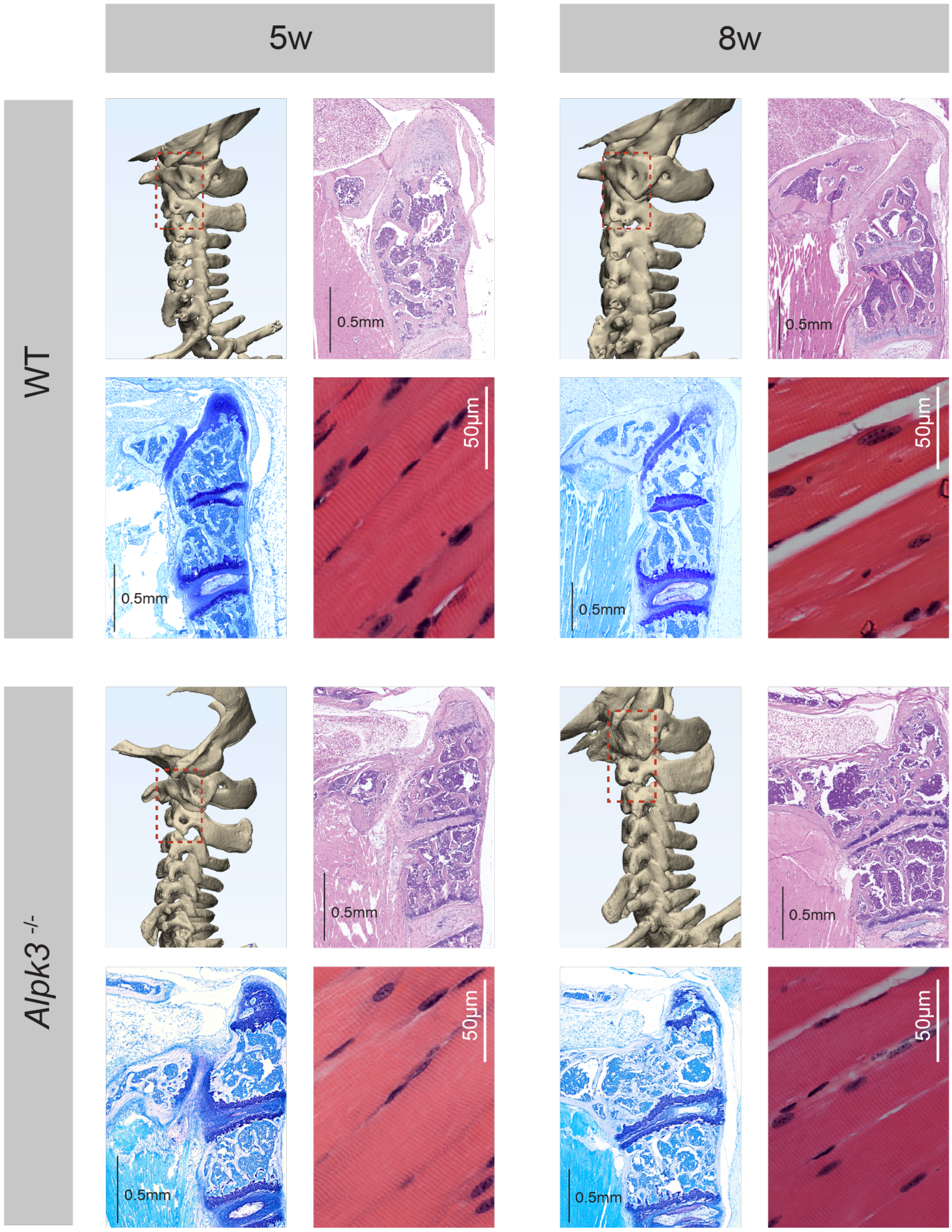

Supplementary Fig. 4 Pathological analyses of cervical vertebrae from *Alpk3*$^{-/-}$ and WT mouse at different ages

MicroCT and cervical vertebrae pathology of *Alpk3*$^{-/-}$ or WT mouse at 5w or 8w. MicroCT, H&E staining of sagittal sections of cervical vertebrae, Toluidine blue staining of sagittal sections of cervical vertebrae, and H&E staining of paraspinal muscle are shown. Note the initiation of abnormal chondrogenesis in *Alpk3*$^{-/-}$ mouse at w5 and bony fusion of C1/C2 in *Alpk3*$^{-/-}$ mouse at w8. No obvious structural anomalies of the skeletal muscle were observed.

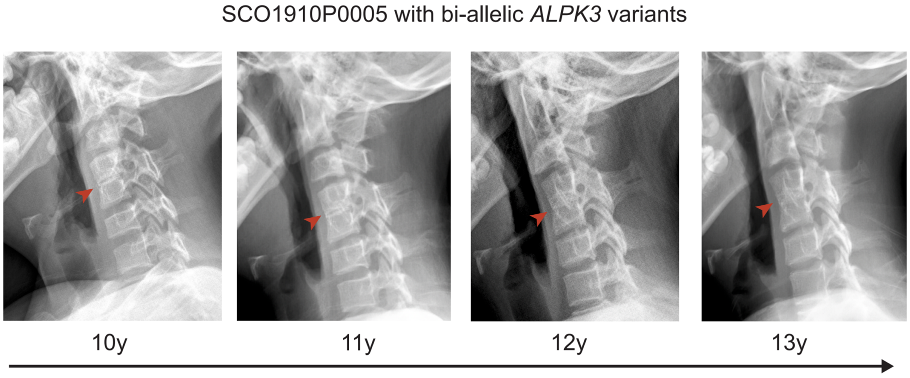

Supplementary Fig. 5 The progressive fusion of cervical vertebrae in SCO1910P0005 with bi-allelic *ALPK3* variants

Lateral cervical spine X-ray of SCO1910P0005 with bi-allelic *ALPK3* variants. The patient was followed up at ages 10y, 11y, 12y, and 13y. Arrowheads indicate the site of vertebral fusion.

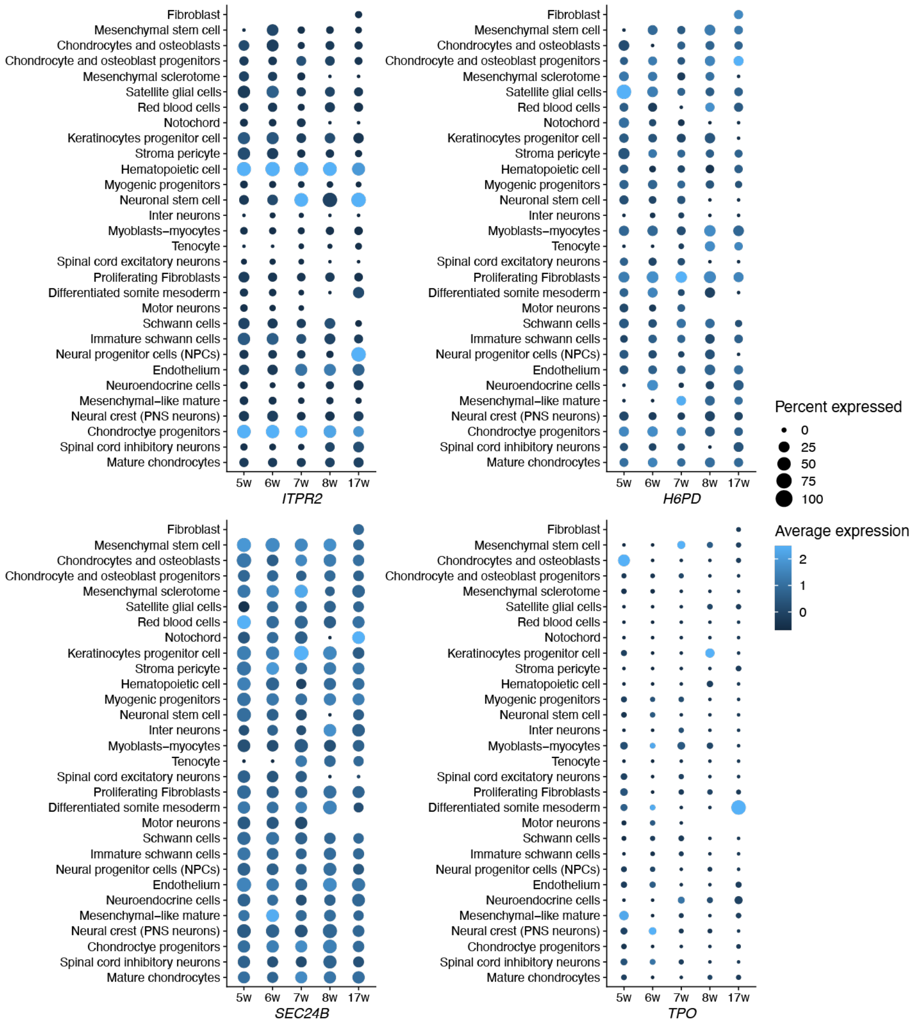

Supplementary Fig. 6 Expression of top burden signals in the developing spine

Expression of four top burden signals (*ITPR2*, *H6PD*, *SEC24B*, *TPO*) in different cell types in human embryonic spine along gestation stages. Percent expressed indicates the percent of cells expressing the gene in each cell type. The average expression indicates the mean normalized gene expression in each cell type.

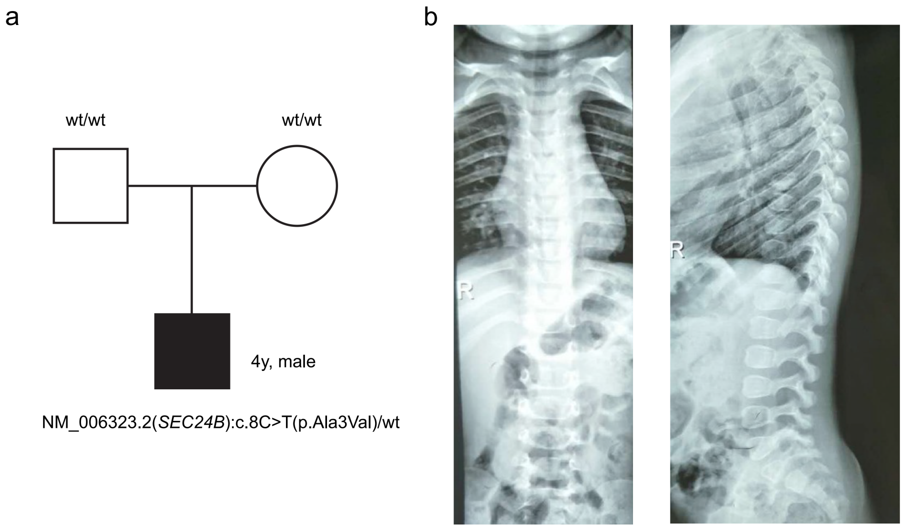

Supplementary Fig. 7 A *de novo* missense variant in *SEC24B*

a, A *de novo* missense variant in *SEC24B* identified from a trio family. **b**, The spinal X-ray images of the proband show lordosis and spina bifida.

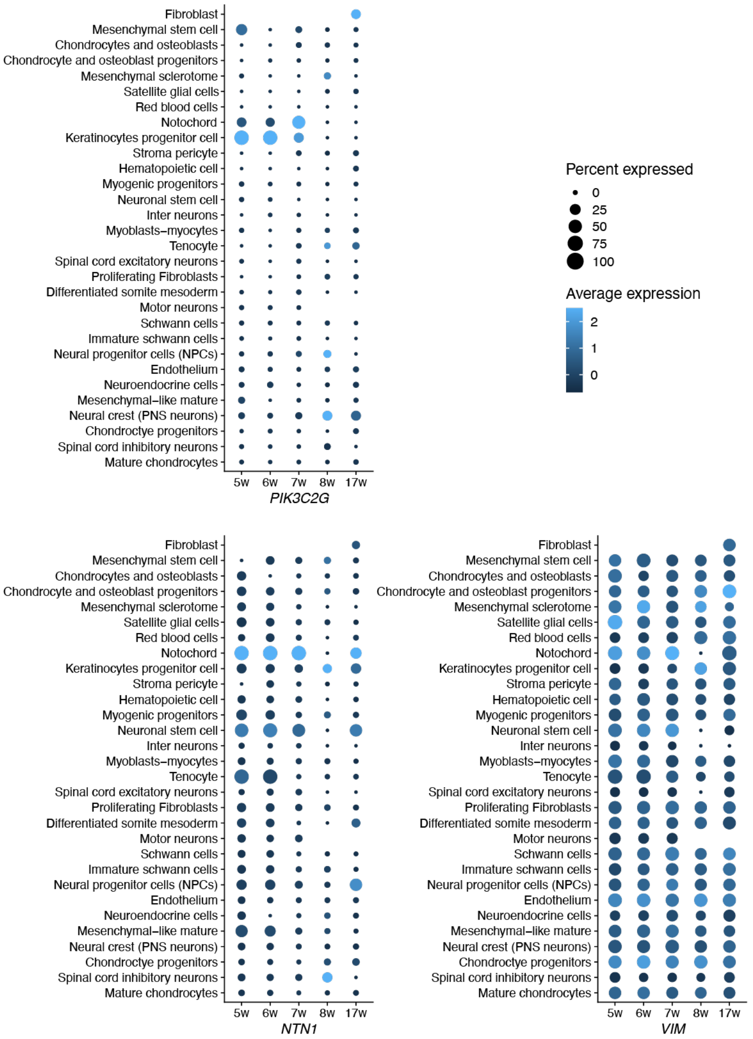

Supplementary Fig. 8 Expression of three notochord-associated genes in the developing spine

Expression of three notochord-associated genes (*PIK3C2G*, *NTN1*, and *VIM*) from burden signals in different cell types in the human embryonic spine along gestation stages. Percent expressed indicates the percent of cells expressing the gene in each cell type. The average expression indicates the mean normalized gene expression in each cell type.

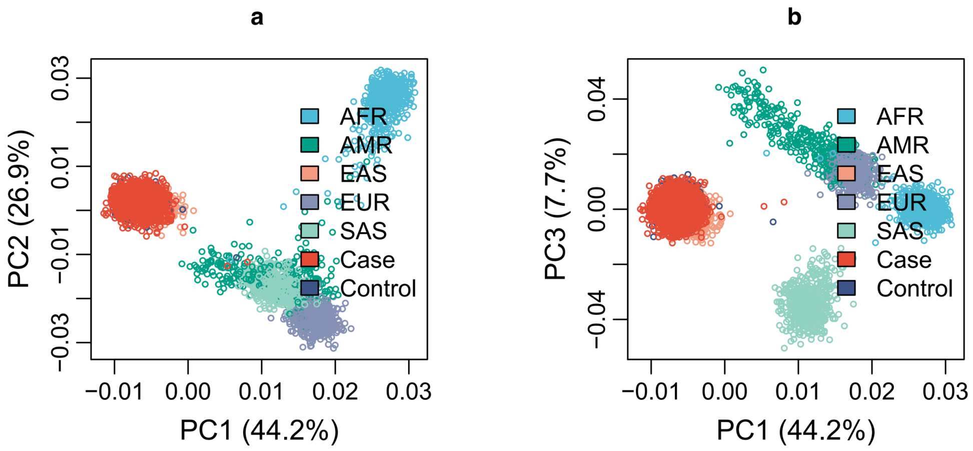

Supplementary Fig. 9 Principal component analysis of the case-control cohort

An ancestry estimation by principal component [14] analysis was performed based on the combined genotypes of the in-house subjects and the 1000 Genomes Phase III population including African (AFR), Admixed American (AMR), East-Asian (EAS), European and South-Asian (SAS). The scatter diagrams are generated based on PC1-PC2 (a) and PC1-PC3 (b) correlations.

Supplementary References

1 Li, H. & Durbin, R. Fast and accurate short read alignment with Burrows-Wheeler transform. *Bioinformatics* **25**, 1754-1760 (2009). https://doi.org:10.1093/bioinformatics/btp324

2 Aldana, R. & Freed, D. Data Processing and Germline Variant Calling with the Sentieon Pipeline. *Methods Mol Biol* **2493**, 1-19 (2022). https://doi.org:10.1007/978-1-0716-2293-3_1

3 McLaren, W. *et al.* The Ensembl Variant Effect Predictor. *Genome Biol* **17**, 122 (2016). https://doi.org:10.1186/s13059-016-0974-4

4 Liu, X., Li, C., Mou, C., Dong, Y. & Tu, Y. dbNSFP v4: a comprehensive database of transcript-specific functional predictions and annotations for human nonsynonymous and splice-site SNVs. *Genome Med* **12**, 103 (2020). https://doi.org:10.1186/s13073-020-00803-9

5 Richards, S. *et al.* Standards and guidelines for the interpretation of sequence variants: a joint consensus recommendation of the American College of Medical Genetics and Genomics and the Association for Molecular Pathology. *Genet Med* **17**, 405-424 (2015). https://doi.org:10.1038/gim.2015.30

6 Fromer, M. & Purcell, S. M. Using XHMM Software to Detect Copy Number Variation in Whole-Exome Sequencing Data. *Curr Protoc Hum Genet* **81**, 7 23 21-21 (2014). https://doi.org:10.1002/0471142905.hg0723s81

7 Abyzov, A., Urban, A. E., Snyder, M. & Gerstein, M. CNVnator: an approach to discover, genotype, and characterize typical and atypical CNVs from family and population genome sequencing. *Genome Res* **21**, 974-984 (2011). https://doi.org:10.1101/gr.114876.110

8 Ruderfer, D. M. *et al.* Patterns of genic intolerance of rare copy number variation in 59,898 human exomes. *Nat Genet* **48**, 1107-1111 (2016). https://doi.org:10.1038/ng.3638

9 Trost, B. *et al.* A Comprehensive Workflow for Read Depth-Based Identification of Copy-Number Variation from Whole-Genome Sequence Data. *Am J Hum Genet* **102**, 142-155 (2018). https://doi.org:10.1016/j.ajhg.2017.12.007

10 Geoffroy, V. *et al.* AnnotSV: an integrated tool for structural variations annotation. *Bioinformatics* **34**, 3572-3574 (2018). https://doi.org:10.1093/bioinformatics/bty304

11 Geoffroy, V. *et al.* AnnotSV and knotAnnotSV: a web server for human structural variations annotations, ranking and analysis. *Nucleic Acids Res* **49**, W21-W28 (2021). https://doi.org:10.1093/nar/gkab402

12 Firth, H. V. *et al.* DECIPHER: Database of Chromosomal Imbalance and Phenotype in Humans Using Ensembl Resources. *Am J Hum Genet* **84**, 524-533 (2009). https://doi.org:10.1016/j.ajhg.2009.03.010

13 Hao, Y. *et al.* Integrated analysis of multimodal single-cell data. *Cell* **184**, 3573-3587 e3529 (2021). https://doi.org:10.1016/j.cell.2021.04.048

14 Gray, R. S. *et al.* Loss of col8a1a function during zebrafish embryogenesis results in congenital vertebral malformations. *Dev Biol* **386**, 72-85 (2014). https://doi.org:10.1016/j.ydbio.2013.11.028
