# Supplementary material for: Unraveling the genetic architecture of congenital vertebral malformation with reference to the developing spine
Source: Nat Commun. 2024 Feb 6;15:1125. doi: 10.1038/s41467-024-45442-5 (PMC10847475; doi:10.1038/s41467-024-45442-5)
Supplement: Supplementary file 3 — Description of Additional Supplementary Files [file 41467_2024_45442_MOESM3_ESM.pdf]

Description of Additional Supplementary Files

File Name: Supplementary Data 1
Description: 16p11.2 deletion in patients diagnosed with TBX6-associated congenital scoliosis (TACS)

File Name: Supplementary Data 2
Description: *TBX6 variants in patients diagnosed with TBX6-associated congenital scoliosis (TACS)*

File Name: Supplementary Data 3
Description: Molecular diagnoses in patients with Mendelian disorders associated with vertebral malformation

File Name: Supplementary Data 4
Description: Pathogenic genomic deletions in CVM patients with syndromic phenotypes

File Name: Supplementary Data 5
Description: Muscular phenotypes in patients with pathogenic variants in muscular disorder associated genes

File Name: Supplementary Data 6
Description: Gene-based burden analysis of ultra-rare variants in 744 molecularly undiagnosed patients against 3740 in-house controls

File Name: Supplementary Data 7
Description: Ultra-rare variants in top five genes in burden analysis

File Name: Supplementary Data 8
Description: Gene markers of each cell cluster from the single-cell transcriptome of human embryonic spine
