# Supplementary material for: Unraveling the genetic architecture of congenital vertebral malformation with reference to the developing spine
Source: Nat Commun. 2024 Feb 6;15:1125. doi: 10.1038/s41467-024-45442-5 (PMC10847475; doi:10.1038/s41467-024-45442-5)
Supplement: Supplementary file 12 — Reporting Summary [file 41467_2024_45442_MOESM12_ESM.pdf]

nature portfolio

Corresponding author(s): Nan Wu, Jianguo Zhang, Zhihong Wu

Last updated by author(s): Jan 3, 2024

Reporting Summary

Nature Portfolio wishes to improve the reproducibility of the work that we publish. This form provides structure for consistency and transparency in reporting. For further information on Nature Portfolio policies, see our Editorial Policies and the Editorial Policy Checklist.

Statistics

For all statistical analyses, confirm that the following items are present in the figure legend, table legend, main text, or Methods section.

n/a	Confirmed	
☐	☒	The exact sample size (*n*) for each experimental group/condition, given as a discrete number and unit of measurement
☐	☒	A statement on whether measurements were taken from distinct samples or whether the same sample was measured repeatedly
☐	☒	The statistical test(s) used AND whether they are one- or two-sided<br>*Only common tests should be described solely by name; describe more complex techniques in the Methods section.*
☐	☒	A description of all covariates tested
☐	☒	A description of any assumptions or corrections, such as tests of normality and adjustment for multiple comparisons
☐	☒	A full description of the statistical parameters including central tendency (e.g. means) or other basic estimates (e.g. regression coefficient) AND variation (e.g. standard deviation) or associated estimates of uncertainty (e.g. confidence intervals)
☐	☒	For null hypothesis testing, the test statistic (e.g. *F*, *t*, *r*) with confidence intervals, effect sizes, degrees of freedom and *P* value noted<br>*Give P values as exact values whenever suitable.*
☒	☐	For Bayesian analysis, information on the choice of priors and Markov chain Monte Carlo settings
☒	☐	For hierarchical and complex designs, identification of the appropriate level for tests and full reporting of outcomes
☒	☐	Estimates of effect sizes (e.g. Cohen's *d*, Pearson's *r*), indicating how they were calculated

Our web collection on statistics for biologists contains articles on many of the points above.

Software and code

Policy information about availability of computer code

Data collection	No software was used for data collection.
Data analysis	Softwares utilized in this study are R v3.6 (https://www.r-project.org/); Python v3.8(https://www.python.org/downloads/); Sentieon v202010 (https://github.com/Sentieon); ACAT R package v0.91 (https://github.com/yaowuliu/ACAT); clusterProfiler R package v4.0 (https://github.com/YuLab-SMU/clusterProfiler); Cellranger v7.0.0 (https://github.com/10XGenomics/cellranger); Seurat v4.2 (https://satijalab.org/seurat/); EWCE R package v1.6 (https://github.com/NathanSkene/EWCE);Variant effect predictor v105 (https://useast.ensembl.org/info/docs/tools/vep/index.html). The codes used for burden analysis are deposited on Github (github.com/zhq921/Burden-pipeline).

For manuscripts utilizing custom algorithms or software that are central to the research but not yet described in published literature, software must be made available to editors and reviewers. We strongly encourage code deposition in a community repository (e.g. GitHub). See the Nature Portfolio guidelines for submitting code & software for further information.

Data

Policy information about availability of data

All manuscripts must include a data availability statement. This statement should provide the following information, where applicable:

- Accession codes, unique identifiers, or web links for publicly available datasets
- A description of any restrictions on data availability
- For clinical datasets or third party data, please ensure that the statement adheres to our policy

The raw genetic sequencing data for CVM patients and control individuals generated in this study have been deposited in the Genome Sequence Archive (GSA, https://ngdc.cncb.ac.cn/gsa-human/) under accession numbers HRA006007 (https://bigd.big.ac.cn/gsa-human/browse/HRA006007) and HRA006052 (https://bigd.big.ac.cn/gsa-human/browse/HRA006052). Raw snRNAseq data generated in this study have been deposited in the GSA under the accession number HRA006073 (https://bigd.big.ac.cn/gsa-human/browse/HRA006073). All raw sequencing data deposited in GSA are under restricted access and only academic use would be approved. A response would be expected within a week. The reference genome used in this study is genome assembly GRCh37/hg19 (https://www.ncbi.nlm.nih.gov/datasets/genome/GCF_000001405.13/). Public data repositories employed throughout this paper include GENCODE (hg19, https://www.gencodegenes.org/human/release_39.html), Ensembl (hg19, https://useast. ensembl.org/index.html), and Genome Aggregation Database (gnomAD v2.1.1, https://gnomad.broadinstitute.org/). Source data are provided with this paper.

Research involving human participants, their data, or biological material

Policy information about studies with human participants or human data. See also policy information about sex, gender (identity/presentation), and sexual orientation and race, ethnicity and racism.

Reporting on sex and gender	Sex was not considered in the study design. The sex of the participants was determined based on self-report and validated through genetic data. No sex-based genetic association analysis was performed because CVM is not a sex-biased disease and we do not have sufficient power for sex-specific analyses.
Reporting on race, ethnicity, or other socially relevant groupings	The participants in this study were all of Asian ancestry. The ethnic identity was self-reported by the participants and validated through genetic data.
Population characteristics	Clinical characteristics of CVM cohort are depicted in Table 1.
Recruitment	We consecutively enrolled Chinese individuals (families) affected with congenital vertebral malformation (CVM) who underwent spinal surgery at Peking Union Medical College Hospital from November 2012 to November 2021 for correction of scoliosis or kyphosis, under the framework of Deciphering disorders Involving Scoliosis and COmorbidities (DISCO) study. All individuals underwent a physical examination, spinal X-ray, spinal computed tomography, spinal magnetic resonance imaging, echocardiography, and renal ultrasound. The inclusion criteria was the presence CVM, which was confirmed by both a radiologist and an orthopedic surgeon. The control cohort was aggregated from individuals who underwent exome sequencing at Peking Union Medical College Hospital, for clinical or research purposes. Individuals recorded to have skeletal malformation or other congenital anomalies were excluded. The retained control cohort includes 3794 unrelated individuals. Written informed consent was obtained at enrollment.<br>The recruitment of patient was performed in the clinic with full consent.<br>Our cohort was skewed toward patients exhibiting severe scoliosis or kyphosis, conditions necessitating surgical intervention.
Ethics oversight	The human genetic study was approved by the Ethics Committee of Peking Union Medical College Hospital (JS-908). Written informed consent was obtained from all participants. Participants were not provided with financial compensation in this study.<br>The collection of human embryos underwent a rigorous ethical review process and was approved by the Ethics Committee of Peking Union Medical College Hospital (I-22PJ819). We provided all donors with comprehensive information about the nature, purpose, and potential outcomes of the research, ensuring that they were fully aware of and understood the aims of our study. We ensured that every donor signed a voluntary consent before any embryo collection occurred during their legally approved pregnancy termination. Donors were not provided with financial compensation in this study. All procedures were carried out strictly following the guidelines of 'Management of Human Genetic Resources', as stipulated by the Ministry of Science and Technology of China (no. 717, effective from July 1, 2019). Detailed records of the embryo acquisition, consent verification, and ethical review were securely maintained by the research team.

Note that full information on the approval of the study protocol must also be provided in the manuscript.

Field-specific reporting

Please select the one below that is the best fit for your research. If you are not sure, read the appropriate sections before making your selection.

☒ Life sciences ☐ Behavioural & social sciences ☐ Ecological, evolutionary & environmental sciences

For a reference copy of the document with all sections, see nature.com/documents/nr-reporting-summary-flat.pdf

Life sciences study design

All studies must disclose on these points even when the disclosure is negative.

Sample size	For human genetics study, all samples available were of Asian ancestry. Participants were excluded if they failed to meet the case and control definitions.<br>For mice and zebrafish studies, no sample size calculation was performed. Sample size was chosen empirically.
Data exclusions	Sequencing data with low quality were excluded in this study. The relevant criteria are detailed in the supplementary Materials and Methods.
Replication	For human genetic study, there was no replication because no other CVM cohort was accessible to us at the time of data freeze.<br>For animal studies, biological replicates were performed independently. The numbers of biological replicates for each experiment are provided in the Methods.
Randomization	Randomization is not relevant for the genetic study which is a retrospective analysis between CVM patients and control. Our study was designed to identify enrichment of rare mutations in genes by comparing observed number of mutations to the expectation independent of ethnic, background, sex, and consanguinity. No intervention was involved.<br>Similarly, randomization is not applicable for our our animal studies, where animals are grouped according to the genotype, and no intervention was involved.
Blinding	Blinding is not relevant for the genetic study because No blinding of patient data was needed given that all patients enter the study with unknown genetic status. All patients analyzed as a whole in the discovery cohort. No validation and test cohort was involved. No prior assumption was made.<br>For animal studies, investigators were blinded to group allocation during data collection and analysis.

Reporting for specific materials, systems and methods

We require information from authors about some types of materials, experimental systems and methods used in many studies. Here, indicate whether each material, system or method listed is relevant to your study. If you are not sure if a list item applies to your research, read the appropriate section before selecting a response.

Materials & experimental systems

n/a	Involved in the study	
☒	☐	Antibodies
☒	☐	Eukaryotic cell lines
☒	☐	Palaeontology and archaeology
☐	☒	Animals and other organisms
☒	☐	Clinical data
☒	☐	Dual use research of concern
☒	☐	Plants

Methods

n/a	Involved in the study	
☒	☐	ChIP-seq
☒	☐	Flow cytometry
☒	☐	MRI-based neuroimaging

Animals and other research organisms

Policy information about studies involving animals; ARRIVE guidelines recommended for reporting animal research, and Sex and Gender in Research

Laboratory animals	C57BL/6 strain was used for mouse experiments, with sterile standard diet and water in a temperature, humidity, and light-controlled rooms. Mice used in the study were housed under controlled environmental conditions. The animal facility maintained a consistent 12-hour light/12-hour dark cycle, with lights on at 07:00 AM and off at 07:00 PM. The ambient temperature in the housing area was consistently maintained at 22 ± 2°C. Relative humidity was maintained at 50 ± 10% throughout the study period. Mice at postnatal day 0 (P0), and at weeks 3 (W3), 5 (W5), 8 (W8), and between 10 to 20 weeks of age (W10-W20) were utilized for experimental procedures.<br>AB/TU strain was used for zebrafish experiments. alpk3a -/- and alpk3b -/- zebrafish strains were crossed and bred to generate the alpk3a/b DKO strain. Zebrafish at 23/24 dpf was utilized for experimental procedures.
Wild animals	This study did not involve wide animals.
Reporting on sex	Sex was not considered in the study design.
Field-collected samples	This study did not involve samples collected from the field.
Ethics oversight	Zebrafish experiments were carried out in compliance with conventional animal handling protocols and received approval from the Animal Care Committee at the Ocean University of China (OU-2012316). Mouse studies were approved by the Ethics Committee of Peking Union Medical College Hospital (XHDW-2022-030).

Note that full information on the approval of the study protocol must also be provided in the manuscript.
